# Supplementary material for: Dirichlet latent modelling enables effective learning and sampling of the functional protein design space
Source: Nat Commun. 2024 Oct 29;15:9309. doi: 10.1038/s41467-024-53622-6 (PMC11519351; doi:10.1038/s41467-024-53622-6)
Supplement: Supplementary file 1 — Supplementary Information [file 41467_2024_53622_MOESM1_ESM.pdf]

Supplementary Figures

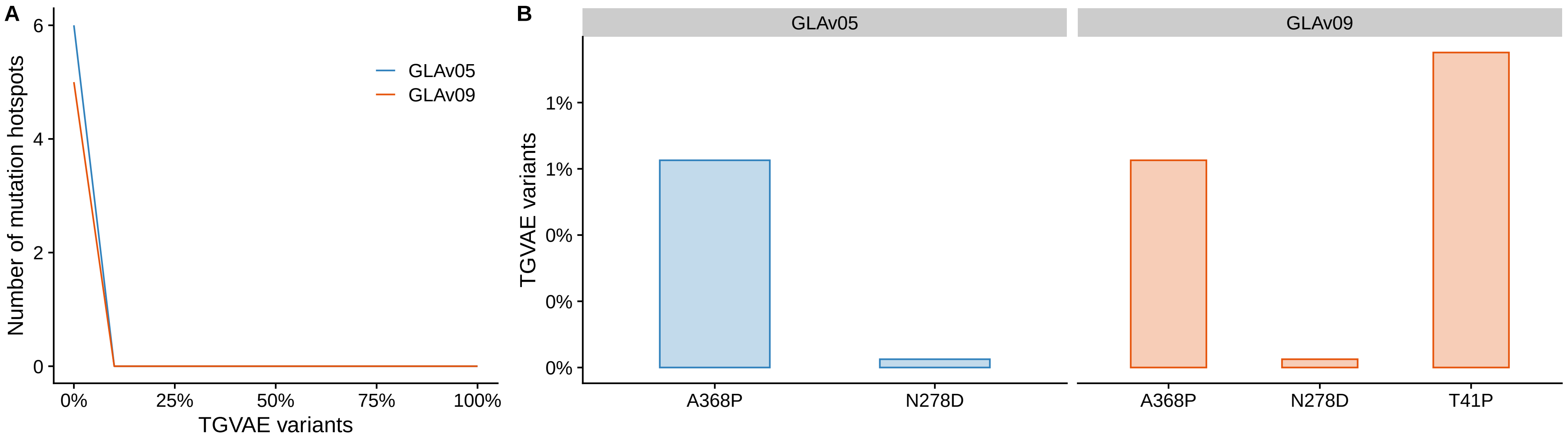

Supplementary Figure 1: Beneficial hotspots and mutations identified by TGVAE. A) Percentage of variants harbouring mutations at the mutational hotspots identified in GLAv05 and GLAv09 [1]. B) Percentage of TGVAE variants carrying GLAv05 and GLAv09 beneficial mutations.

Supplementary References

1. Hallows, W. C. *et al.* Optimizing human α-galactosidase for treatment of Fabry disease. *Scientific Reports* **13,** 4748 (2023).
