# Supplementary material for: Dirichlet latent modelling enables effective learning and sampling of the functional protein design space
Source: Nat Commun. 2024 Oct 29;15:9309. doi: 10.1038/s41467-024-53622-6 (PMC11519351; doi:10.1038/s41467-024-53622-6)
Supplement: Supplementary file 3 — Description of Additional Supplementary Files [file 41467_2024_53622_MOESM3_ESM.pdf]

**Description of Additional Supplementary Files**

Supplementary Data 1:

- **Model comparison** tab: Protein level comparison between TDVAE and DeepSequence on the mutation effect prediction task.
- **Mutagenesis summary performance** tab: Summary correlation performances for each protein for TDVAE and DeepSequence.
- **Mutagenesis raw data** tab: Raw performance data for each protein for TDVAE and DeepSequence.

Supplementary Data 2:

- **Hyperparameter settings** tab: Description of hyperameters settings used in the study.
- **TDVAE vs TGVAE** tab: Comparison for 9 scenarios between TCN-based VAEs with Dirichlet (TDVAE) and Gaussian TGVAE) latent space.
- **YAP1 … BLAT-Tenaillon2013** tabs (8 tabs): Each tab contains the results for the mutation effects prediction task for each of the protein in the benchmark set.
